# Multiplexed-tandem PCR for the specific diagnosis of gastrointestinal nematode infections in sheep: an European validation study

**DOI:** 10.1186/s13071-017-2165-x

**Published:** 2017-05-08

**Authors:** Florian Roeber, Alison Morrison, Stijn Casaert, Lee Smith, Edwin Claerebout, Philip Skuce

**Affiliations:** 1 AusDiagnostics Pty. Ltd, Beaconsfield, 2015 NSW Australia; 2 Moredun Research Institute, Pentlands Science Park, Bush Loan, Penicuik, EH26 0PZ UK; 3 Department of Virology, Parasitology and Immunology, Faculty of Veterinary Medicine, Ghent University, Salisburylaan 133, 9820 Merelbeke, Belgium

**Keywords:** Small ruminants, Sheep, Parasites, Trichostrongylid nematodes, Molecular diagnosis, MT-PCR

## Abstract

**Background:**

Traditional methods of detecting and identifying gastrointestinal nematode infections in small ruminants, including sheep and goats, are time-consuming and lack in sensitivity and specificity. Recently, we developed an automated multiplexed-tandem (MT)-PCR platform for the diagnosis and identification patent infections with key genera/species of gastrointestinal nematodes of sheep and validated this approach in detailed experiments carried out in Australia. In the present study, we deployed this diagnostic platform in Scotland and Belgium to test samples from naturally infected sheep in these environments and to validate the MT-PCR platform relative to traditional diagnostic methods routinely used by local laboratories.

**Results:**

MT-PCR detected all microscopy positive samples and there was a significant agreement between the results of the different test methods in terms of the species detected and their relative proportion in a test sample, however, for some samples, there were discrepancies between the results of the different test methods. Selective sequencing of purified MT-PCR products demonstrated the results to be 100% specific.

**Conclusions:**

The MT-PCR platform is an advanced method for the species/genus-specific diagnosis of gastrointestinal nematode infections in small ruminants and has demonstrated utility when deployed in different countries and climatic zones. The platform is user-friendly due to the largely automated procedure and has high versatility in that it can achieve a specific diagnosis from different types of sample templates, including larval culture and faecal samples. With appropriate modifications of the primers used, the MT-PCR platform also provides potential for the diagnosis of a variety of other pathogens of veterinary or medical importance.

**Electronic supplementary material:**

The online version of this article (doi:10.1186/s13071-017-2165-x) contains supplementary material, which is available to authorized users.

## Background

Gastrointestinal nematodes (belonging to the Order Strongylida) frequently infect small ruminants (e.g. sheep and goats) and represent a common and costly problem to the livestock industry around the world [1, 2]. Infections with these parasites result in morbidity and mortality of their hosts and incur additional costs due to anthelmintic treatments used to control disease caused by these parasites [3, 4]. Traditional methods of diagnosis, including faecal egg counts (FEC) and larval culture (LC), lack sensitivity (ability to detect truly infected animals; false negative test results) and specificity (ability to correctly identify the parasite species; false positives) and can be time-consuming to conduct [5]. They also require experienced personnel for accurate species identification, as many trichostrongylid eggs and larvae are difficult to distinguish morphologically [6]. Therefore, there is a need for the development of improved diagnostic methods that can be relatively easily adopted by diagnostic testing laboratories.

Recently, an automated multiplexed-tandem polymerase chain reaction (MT-PCR) platform for diagnosis of the most important genera and species of sheep gastrointestinal nematodes, including *Teladorsagia circumcincta*, *Haemonchus contortus*, *Trichostrongylus* spp., *Chabertia ovina*, *Oesophagostomum* spp. (including *O. venulosum* and *O. columbianum*) and *Cooperia curticei* has been developed and extensively tested and validated in Australia [7]. This method is based on the detection and enzymatic amplification of species-specific markers of nuclear ribosomal deoxyribonucleic acid (DNA). In our previous studies conducted in Australia, this novel molecular diagnostic approach demonstrated to be highly sensitive and specific in the context of diagnosing naturally infected sheep. It also showed excellent agreement with the results of traditional diagnostic techniques such as FEC, LC and *post-mortem* total worm counts in regards to the presence/absence of different nematode species in naturally infected sheep [7]. Even though the sequences of the second internal transcribed spacer (ITS2), which are used during the molecular diagnostic process are highly conserved, some variation between and/or within different populations of parasites may occur, especially from different geographical locations [8, 9]. Therefore, in this proof-of-concept study, our aim was to deploy this new MT-PCR approach to the European environment and to determine its performance on samples obtained from sheep in Scotland and Belgium. Furthermore, we used samples from these countries to compare the results of this MT-PCR approach with the results of FEC and LC as performed by the relevant participating laboratories.

## Methods

### Sample collection and routine coprological methods

The Moredun Research Institute, Scotland, and the Laboratory of Parasitology at the Veterinary Faculty, Ghent University, Belgium, participated in this study. Fresh individual faecal samples were obtained locally from naturally infected sheep. Both laboratories performed faecal flotation, nematode egg counting and LC according to their respective routine protocols. At Moredun, Scotland, a total of 11 faecal samples were collected from naturally infected sheep during February 2016. Faecal egg counts were performed using a modified salt flotation method as described by Christie & Jackson [10], with a sensitivity of 1 egg per gram of faeces (epg). LCs were carried out using an incubation of 22 °C for a period of 10–14 days. A modified Baermann technique [11] was used for the recovery of third-stage larvae from culture samples. In brief, sample pots were flooded with tepid water and left to stand for 4 h, the resultant water was then poured off the faeces and used to pass over a jam jar sized mini Baermann apparatus set up with 3 ply nappy liner paper. Once all the water has been passed over the paper this was then placed over the top of a jam jar and allowed to soak overnight in tepid water. Larvae were recovered the following day from the bottom of the jar after syphoning off the supernatant. In Ghent, a total number of 8 faecal samples from naturally infected sheep were collected between June-October 2015. Sample aliquots intended for the enumeration of nematode eggs were stored refrigerated (4 °C) for a maximum period of 11 days before microscopic examination. Faecal egg counts were performed by a modified McMaster technique using 4 g of faecal matter for the enumeration of eggs and with a theoretical detection limit of 50 epg [11]. For the preparation of LCs, 20–30 g of fresh faecal material was plated out on a moist paper filter in a Petri dish. Faecal cultures were incubated at 25 °C for 10–14 days, larvae were recovered via Baermannisation and identification of third-stage larvae, which was based on the morphological key as given by van Wyk et al. [12].

### Egg harvest, genomic DNA extraction and MT-PCR set-up

The automated MT-PCR diagnostic platform (*Easy-Plex*, AusDiagnostics Pty. Ltd., Beaconsfield, Australia) was installed at both participating laboratories and the relevant operators trained in the steps of DNA extraction from nematode egg and/or larval culture samples, set-up of MT-PCR reactions and analysis of results. The steps of separating nematode eggs from bulk faecal matter, DNA extraction and MT-PCR set-up were carried out in both laboratories according to the procedure described by Roeber et al. [7]. For every faecal sample tested, 4 g of faeces was suspended in 60 ml of saturated saline in a screw cap pot (Sarstedt Ltd). The suspension was strained through a tea-sieve to remove larger faecal debris and 50 ml were transferred to a Falcon conical tube and centrifuged at 1,000× *g* for 2 min. Five ml of the supernatant (containing the eggs) was decanted into a fresh tube and the volume increased to 50 ml by adding water. The suspension was centrifuged one more time at 2,000× *g* for 5 min. The supernatant was discarded without disturbing the faecal pellet in the bottom of the tube. The pellet was harvested into a 2 ml Eppendorf tube, and stored at -20 °C in a freezer or used directly for DNA extraction. The pellet was spun at 1,000× *g* for 1 min and a 250 μl aliquot of the precipitate used for DNA extraction using a commercial kit (PowerSoil® DNA Isolation Kit, Mobio Laboratories Inc., USA) following the manufacturer’s instructions and eluted in 100 μl molecular-grade water. For the amplification of nematode DNA from eggs, 5 μl of genomic DNA sample was used during the set-up of the MT-PCR. Additionally, from every LC sample prepared at the Ghent (*n* = 8) and Moredun laboratory (*n* = 11), 250 μl of larval culture sample were used for DNA extraction and subsequent MT-PCR using the same protocol as for nematode egg samples.

### Robotic reaction set-up and MT-PCR assays

At both laboratories, MT-PCR was performed using the Easy-Plex system (AusDiagnostics Pty. Ltd., Australia), consisting of a Rotor-Gene 6000 real-time PCR thermocycler (Qiagen, Hilden, Germany), and a Gene-Plex CAS1212 liquid handling robot (AusDiagnostics Pty. Ltd.). Specific primers (AusDiagnostics Pty. Ltd.) were designed to the internal regions of the ITS2 sequences of *Haemonchus* spp. (*H. contortus* and *H. placei*), *Teladorsagia circumcincta*, *Trichostrongylus* spp., *Oesophagostomum* spp. *(O. columbianum* and *O. venulosum*; combined assay) and *Chabertia ovina* in order to produce amplicons of 100–200 bp (depending on species) in the second phase of MT-PCR. For the amplification of *Cooperia curticei*, a specific sequence in the ddASP gene was used for the primer design because the ITS2 sequence did not provide a species-specific sequence tag for this species and/or cross-reacted with the other assays designed. Additionally, a ‘pan-nematode’ assay was developed, specific to the ITS2 sequences of nematodes and included as a positive control. For the amplification of samples during each MT-PCR run, 5 μl of genomic DNA representing each sample (*n* = 7, plus one no-template (negative) control) were loaded into 0.2 ml PCR strips and placed into a 24-well thermocycling block within the Gene-Plex robotic platform. Following the loading of each sample and the initiation of the ‘Sheep Parasites Assay’, the ‘medium sensitivity’ setting (15 cycles in Step 1 PCR) was selected and the remainder of the set-up process and analysis was directed by the program ‘Easy-Plex Assay Setup’ (AusDiagnostics Pty. Ltd.), with all of the remaining steps of the MT-PCR procedure being semi-automated [13]. A sample was recorded as test-positive using the ‘auto-call function’ of the Easy-Plex software, if the amplicon produced a single melting curve which was within 1.5 °C of the expected melting temperature, the height of the peak was higher than 0.2 dF/dT and the peak width was ≤ 3.8 °C (AusDiagnostics Pty. Ltd.). Cycle threshold (Ct) values were recorded for each test-positive sample, and quantitative values for each parasite in each sample were determined using an automated comparison with Ct data determined for an internal spike-control (tube containing 10,000 copies of a synthesized oligonucleotide template and a specific primer set) for each sample tested [13]. Samples that produced a very early amplification (i.e. < 10 cycles) were diluted either at 1/50 or 1/100 and re-tested.

### Statistical analysis of results

To directly compare the results of MT-PCR and LC, the following modifications were made to the dataset. The quantitative results (gene copy number values) as recorded by MT-PCR for *Co. curticei* were multiplied by 100 to make the results for this species directly comparable with the results of the other species MT-PCR assays, which are based on the amplification of the ITS2 locus of nuclear ribosomal DNA. The rationale for this is that the ddASP locus used for the detection of *Co. curticei* is a single copy gene (i.e. it only occurs once in the genome of the parasite) whereas the ITS2 locus used for the design of the other species assays is predicted (inferred from the *H. contortus* genome [14, 15]) to be represented ~100 times in the genome of this parasite, and was thus used as a reference for the adjustment of *Co. curticei* gene copy numbers as determined by the ddASP-based MT-PCR assay.

To compare the MT-PCR results with the percentage values determined by LC, the quantitative results recorded following MT-PCR for each of the six species/genera were added together for each sample to calculate the total amount of detected DNA copies per sample. The result recorded for an individual species was then divided by the total amount of DNA detected per sample and multiplied by 100.

Furthermore, because it is very difficult and unreliable to morphologically differentiate the long-tailed larvae of *C. ovina* and *Oesophagostomum* spp., no attempt at differentiation was made. Also, the specific MT-PCR results for *C. ovina* and *Oesophagostomum* spp. were combined to make the datasets directly comparable.

The percentage results, as determined by MT-PCR as well as by LC, were plotted in a stacked column chart using Microsoft Excel 2010. To determine the agreement between the two techniques (i.e. MT-PCR versus LC) to detect each nematode species, pivot tables were created in the Microsoft Excel 2010 software package and the level of agreement as well as Kappa values were determined using an established approach [16], using the WinEpiscope online tool (http://www.winepi.net/). Interpretation of Kappa was done according to the definitions of Landis and Koch [17]. Kappa values were adjusted for bias and prevalence (PABAK) according to Watson & Petrie [18].

## Results

**Fig. 1:**
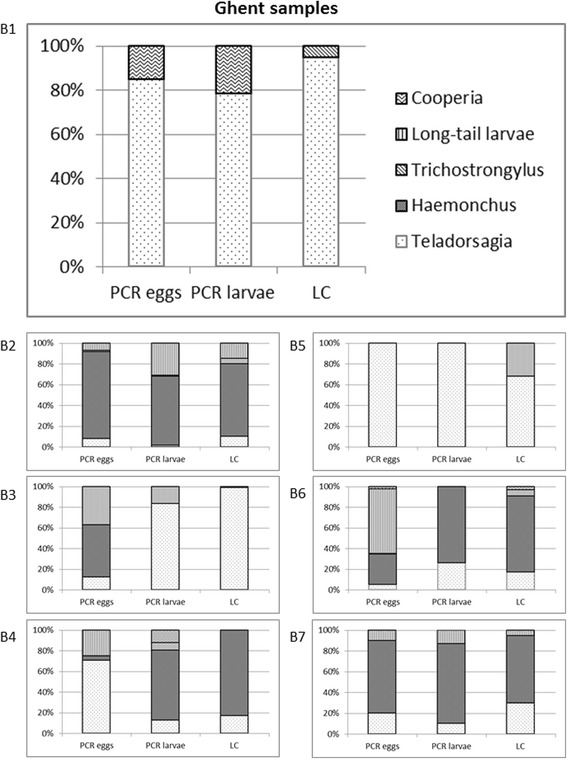
Percentage results for the species/genera of *T. circumcincta*, *Haemonchus* spp., *Trichostrongylus* spp., *Co. curticei* and the combined results for *C. ovina* and *Oesophagostomum* spp. as determined by the three different diagnostic methods used and for each sample. Shown are the results for the Belgian samples B1-B7. An additional file shows the results for all samples (see Additional file 1)

**Fig. 2:**
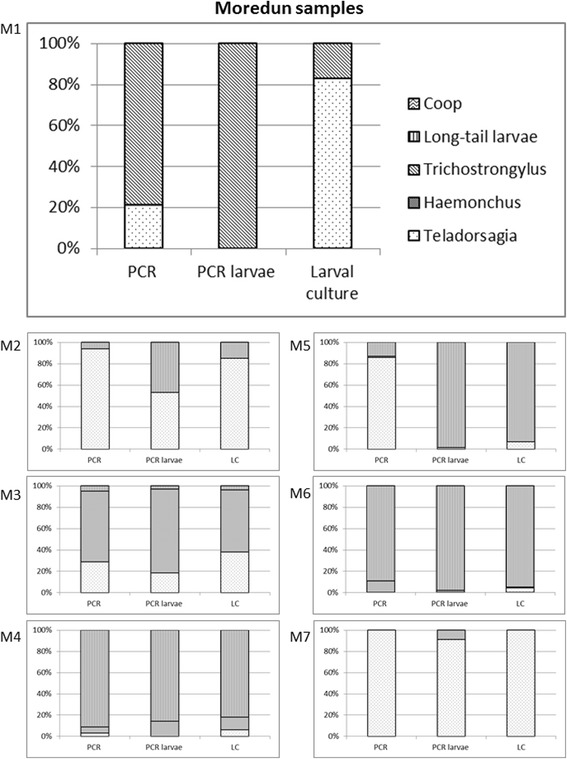
Percentage results for the species/genera of *T. circumcincta*, *Haemonchus* spp., *Trichostrongylus* spp., *Co. curticei* and the combined results for *C. ovina* and *Oesophagostomum* spp. as determined by the three different diagnostic methods used and for each sample. Shown are the results for the Scottish samples M1-M7. An additional file shows the results for all samples (see Additional file 1)

At both laboratories, all samples tested returned positive results by egg count (range 2–1,713 epg) and MT-PCR. Every sample tested was analysed by three different methods, which were: MT-PCR eggs (MT-PCR_(e)_), MT-PCR larvae (MT-PCR_(l)_) and LC. There was a significant agreement between the results of MT-PCR (eggs and/or larvae) and LC technique for the species detected as well as their determined proportions in an egg and/or LC sample (Table 1). In most samples, the results of all three tests employed (i.e. MT-PCR_(e)_, MT-PCR_(l)_ and LC) consistently determined the species/genus with the highest proportion in a particular sample. *Teladorsagia. circumcincta* was consistently identified by all three tests to have the highest proportion in samples M2, M7, M8, M9, B1, and sample B5 (Figs. 1–2; see Additional file 1: Figure S1). *Haemonchus contortus* was identified by all three tests to have the highest proportion in samples M11, B2 and B7. In contrast, *Trichostrongylus* spp. predominated in sample M3 and *C. ovina* and/or *Oesophagostomum* in sample M4 and M6 (Figs. 1–2; see Additional file 1: Figure S1). For other samples, there was some disagreement between the results of the respective diagnostic approaches. This applied to samples M1, M5, M10, B3, B4, B6 and B8 (Table 1). In each of these samples the results of all three diagnostic methods differed in terms of either the species detected or in terms of the proportion as determined for certain species by the different test methods (Table 1).

**Table 1:** Results of samples collected at Moredun, Scotland and Ghent, Belgium and tested by three different methods; multiplexed-tandem (MT)-PCR eggs (MT-PCR_(e)_), MT-PCR larvae (MT-PCR_(l)_) and larval culture (LC)

			MT-PCR gene copy number (calculated percentage)					
Sample id (location)	EPG	Test	Tel	Haem	Trich	Cho	Oeso	Coc
M1	585	Mt-PCR_(e)_^a^	693 (21)	0 (0)	2,572 (79)*	0 (0)	0 (0)	0 (0)
(Moredun)		MT-PCR_(l)_^a^	0 (0)	0 (0)	120 (100)*	0 (0)	0 (0)	0 (0)
		LC %	83*	0	17	0		0
M2	90	Mt-PCR_(e)_^a^	458 (94)	0 (0)	0 (0)	0 (0)	31 (6)	0 (0)
(Moredun)		MT-PCR_(l)_^a^	2,062 (53)	0 (0)	45 (1)	1,788 (45)	44 (1)	0 (0)
		LC %	85	0	0	15		0
M3	243	Mt-PCR_(e)_^a^	165 (29)	0 (0)	379 (66)	0 (0)	29 (5)	0 (0)
(Moredun)		MT-PCR_(l)_^a^	261 (18)	0 (0)	1,123 (79)	0 (0)	43 (3)	0 (0)
		LC %	38	0	58	4		0
M4	30	Mt-PCR_(e)_^a^	24 (3)	0 (0)	49 (6)	0 (0)	772 (91)	0 (0)
(Moredun)		MT-PCR_(l)_^a^	0 (0)	0 (0)	295 (14)	0 (0)	1,799 (86)	0 (0)
		LC %	6	0	12	82		0
M5	176	Mt-PCR_(e)_^a^	1,997 (86)*	0 (0)	26 (1)	309 (13)	0 (0)	0 (0)
(Moredun)		MT-PCR_(l)_^a^	0 (0)	0 (0)	33 (1)	2,503 (99)*	0 (0)	0 (0)
		LC %	7	0	0	93*		0
M6	59	Mt-PCR_(e)_^b^	0 (0)	0 (0)	146 (11)	1,241 (89)	0 (0)	0 (0)
(Moredun)		MT-PCR_(l)_^a^	77 (0.3)	0 (0)	325 (1)	22,566 (99)	0 (0)	0 (0)
		LC %	4	0	1	94		0
M7	13	Mt-PCR_(e)_^b^	113 (100)	0 (0)	0 (0)	0 (0)	0 (0)	0 (0)
(Moredun)		MT-PCR_(l)_^a^	631 (91)	0 (0)	61 (9)	0 (0)	0 (0)	0 (0)
		LC %	100	0	0	0		0
M8	11	Mt-PCR _(e)_	16,693 (100)	0 (0)	0 (0)	0 (0)	0 (0)	0 (0)
(Moredun)		MT-PCR_(l)_^b^	514 (76)	0 (0)	0	165 (24)	0 (0)	0 (0)
		LC %	97	0	0	3		0
M9	171	Mt-PCR_(e)_^b^	3,994 (82)	0 (0)	602 (12)	133 (3)	158 (3)	0 (0)
(Moredun)		MT-PCR_(l)_^a^	1,075 (79)	0 (0)	245 (18)	0 (0)	42 (3)	0 (0)
		LC %	79	0	15	6		0
M10	36	Mt-PCR _(e)_	33,346 (30)	0 (0)	47,858 (43)**	24,912 (22)	2,709 (3)	2,100 (2)
(Moredun)		MT-PCR_(l)_	19 (0.03)	0 (0)	8,189 (14)	42,319 (72)*	0 (0)	8,100 (14)
		LC %	34	0	2	14		50*
M11	720	Mt-PCR _(e)_^b^	358 (1)	27,983 (92)	130 (0.43)	1,792 (6)	253 (1)	0 (0)
(Moredun)		MT-PCR_(l)_^b^	0 (0)	4,309 (99)	62 (1)	0 (0)	0 (0)	0 (0)
		LC %	0 (0)	98	0	1		1
B1	176	Mt-PCR_(e)_	10,762 (85)	15 (0.12)	0 (0)	0 (0)	0 (0)	1,900 (15)
(Ghent)		MT-PCR_(l)_	14,617 (78)	40 (0.21)	0 (0)	0 (0)	0 (0)	4,000 (21)
		LC %	92	0	8	0	0	0
B2	2	Mt-PCR_(e)_	13,157 (8)	135,922 (84)	1,793 (1)	11,398 (7)	0 (0)	0 (0)
(Ghent)		MT-PCR_(l)_	1,619 (1)	79,775 (67)	887 (1)	36,547 (31)	0 (0)	0 (0)
		LC %	10	68	5	17		0
B3	176	Mt-PCR_(e)_	8,262 (12)	34,497 (51)*	25,297 (37)	0 (0)	0 (0)	0 (0)
(Ghent)		MT-PCR_(l)_	3,887 (84)*	0 (0)	752 (16)	0 (0)	0 (0)	0 (0)
		LC %	99*	1	0	0		0
B4	72	Mt-PCR_(e)_	6,914 (71)*	406 (4)	16 (0.16)	2,436 (25)	0 (0)	0 (0)
(Ghent)		MT-PCR_(l)_	4,154 (13)	21,880 (68)*	2,275 (7)	0 (0)	0 (0)	3,900 (12)
		LC %	17	83*	0	0		0
B5	200	Mt-PCR_(e)_	22,039 (100)	0 (0)	0 (0)	0 (0)	0 (0)	0 (0)
(Ghent)		MT-PCR_(l)_	172,336 (100)	0 (0)	0 (0)	0 (0)	0 (0)	0 (0)
		LC %	68	0	32	0		0
B6	986	Mt-PCR_(e)_	6,741 (5)	39,499 (30)	220 (0.17)	83,044 (63)*	0 (0)	3,100 (2)
(Ghent)		MT-PCR_(l)_	175 (26)	494 (74)*	0 (0)	0 (0)	0 (0)	0 (0)
		LC %	17	74*	6	0		3
B7	1584	Mt-PCR_(e)_	153,756 (20)	531,837 (70)	0 (0)	72,565 (10)	945 (0.12)	0 (0)
(Ghent)		MT-PCR_(l)_	1,195,981 (10)	9,266,891 (77)	1,955 (0.02)	1,436,634 (12)	83,435 (1)	5,900 (0.05)
		LC %	30	65	0	5		0
B8	804	Mt-PCR_(e)_	267,862 (11)	39 (0.00)	566,001 (24)	0 (0)	1,414,909 (59)*	156,200 (6)
(Ghent)		MT-PCR_(l)_	8,309 (3)	56 (0.02)	84,321 (28)	0 (0)	28,087 (9)	180,300 (60)*
		LC %	24	1	4	10		61*

^a^Samples tested at 1/50 dilution

^b^Samples tested at 1/100 dilution

*Samples with discrepant results

For every sample, the microscopy results are given as eggs per gram of faeces (EPG). The species/genera-specific results of the MT-PCR for *T. circumcincta* (Tel), *Haemonchus* spp. (Haem), *Trichostrongylus* spp. (Trich), *C. ovina* (Cho), *Oesophagostomum* spp. (Oeso) and *Co. curticei* (Coc) are shown as gene copy number or as calculated percentage (%). Discrepancies between the results of the three diagnostic methods are indicated by an asterisk

The agreement of positive versus negative results between MT-PCR_(e)_ and LC across the whole dataset was highest for *Haemonchus* spp., with a calculated agreement of 94.70%, PABAK 0.895 (almost perfect agreement), followed by *Co. curticei* and *T. circumcincta* with 89.50% agreement and a PABAK of 0.789 (substantial agreement) for both species (Table 1). Agreement between MT-PCR_(e)_ and LC for the species/genera of *C. ovina*/*Oesophagostomum* was 84.20%, PABAK 0.684 (substantial agreement) and lowest for species of *Trichostrongylus* with 68.40% agreement and a PABAK of 0.368 (fair agreement). The agreement of positive versus negative results for MT-PCR_(l)_ and LC was highest for species/genera of *C. ovina*/*Oesophagostomum*, with a calculated agreement of 94.70%, PABAK 0.895 (almost perfect agreement), followed by *Haemonchus* spp. with 89.50% agreement, PABAK 0.789 (substantial agreement), *T. circumcincta* with 84.20% agreement, PABAK 0.684 (substantial agreement), *Co. curticei* with 73.70% agreement, PABAK 0.474 (moderate agreement) and *Trichostrongylus* spp. with 47.40% agreement, PABAK -0.053 (agreement by chance). In general, the agreement between the results of MT-PCR_(e)_versus LC was higher than the agreement between MT-PCR_(l)_versus LC. Also, the results for samples tested at the Moredun laboratory showed higher levels of agreement (for eggs and larvae versus LC) compared to the samples tested at the Ghent laboratory (Table 2). Overall, *T. circumcincta* was the most frequently detected nematode species in samples from Scotland and Belgium (Table 3). *Haemonchus* spp. and *Co. curticei* had higher prevalence in samples from Belgium, whereas *C. ovina*/*Oesophagostomum* spp. were of higher prevalence in samples from Scotland. *Trichostrongylus* spp. were more prevalent in samples from Scotland as determined by MT-PCR (eggs and larvae), whilst its prevalence was slightly higher in Belgium as determined by LC. Representative amplicons (*n* = 17) were sequenced and demonstrated that in every case the particular target sequence has been amplified and that the results obtained are 100% specific.

**Table 2:** Agreement (%) between the results of multiplexed-tandem (MT)-PCR eggs versus larval culture (LC) and MT-PCR larvae (larvae) versus LC

Species	*n*	+,+	+,-	-,+	-,-	Agreement (%)	Kappa	PABAK
MT-PCR		Larval culture - Moredun (22 °C)						
*Te. circumcincta* (MT-PCR _(e)_)	11	9	1	1	0	81.80	-0.100	0.636
*Te. circumcincta* (MT-PCR_(l)_)	11	7	0	3	1	72.70	0.298	0.455
*Haemonchus* (MT-PCR _(e)_)	11	1	0	0	10	100.00	1.000	1.000
*Haemonchus* (MT-PCR_(l)_)	11	1	0	0	10	100.00	1.000	1.000
*Trichostrongylus* (MT-PCR _(e)_)	11	6	2	0	3	81.80	0.620	0.636
*Trichostrongylus* (MT-PCR_(l)_)	11	6	4	0	1	63.60	0.214	0.273
*Chabertia*/*Oesophagostomum* (MT-PCR _(e)_)	11	8	0	1	2	90.90	0.744	0.818
*Chabertia*/*Oesophagostomum* (MT-PCR_(l)_)	11	8	0	1	2	90.90	0.744	0.818
*C. curticei* (MT-PCR _(e)_)	11	1	0	1	9	90.90	0.621	0.818
*C. curticei* (MT-PCR_(l)_)	11	1	0	1	9	90.90	0.621	0.818
		Larval culture - Belgium (25 °C)						
*Te. circumcincta* (MT-PCR _(e)_)	8	8	0	0	0	100.00	-1.000	1.000
*Te. circumcincta* (MT-PCR_(l)_)	8	8	0	0	0	100.00	-1.000	1.000
*Haemonchus* (MT-PCR _(e)_)	8	6	1	0	1	87.50	0.600	0.750
*Haemonchus* (MT-PCR_(l)_)	8	5	1	1	1	75.00	0.330	0.500
*Trichostrongylus* (MT-PCR _(e)_)	8	3	2	2	1	50.00	-0.067	0.000
*Trichostrongylus* (MT-PCR_(l)_)	8	2	3	3	0	25.00	-0.600	-0.500
*Chabertia*/*Oesophagostomum* (MT-PCR _(e)_)	8	3	2	0	3	75.00	0.529	0.500
*Chabertia*/*Oesophagostomum* (MT-PCR_(l)_)	8	3	0	0	5	100.00	1.000	1.000
*C. curticei* (MT-PCR _(e)_)	8	2	1	0	5	87.50	0.714	0.750
*C. curticei* (MT-PCR_(l)_)	8	1	3	1	3	50.00	0.000	0.000
		Larval culture - Whole dataset (22 °C and 25 °C)						
*Te. circumcincta* (MT-PCR _(e)_)	19	17	1	1	0	89.50	-0.056	0.789
*Te. circumcincta* (MT-PCR_(l)_)	19	15	0	3	1	84.20	0.345	0.684
*Haemonchus* (MT-PCR _(e)_)	19	7	1	0	11	94.70	0.890	0.895
*Haemonchus* (MT-PCR_(l)_)	19	6	1	1	11	89.50	0.774	0.789
*Trichostrongylus* (MT-PCR _(e)_)	19	9	4	2	4	68.40	0.329	0.368
*Trichostrongylus* (MT-PCR_(l)_)	19	8	7	3	1	47.40	-0.159	-0.053
*Chabertia*/*Oesophagostomum* (MT-PCR _(e)_)	19	11	2	1	5	84.20	0.650	0.684
*Chabertia*/*Oesophagostomum* (MT-PCR_(l)_)	19	11	0	1	7	94.70	0.890	0.895
*C. curticei* (MT-PCR _(e)_)	19	3	1	1	14	89.50	0.683	0.789
*C. curticei* (MT-PCR_(l)_)	19	2	3	2	12	73.70	0.275	0.474

Also shown are the calculated Kappa values and the numbers of samples tested positive and negative by each method and for each laboratory as well as for the entire dataset

**Table 3:** Results of specific testing of samples by three different diagnostic methods; multiplexed-tandem (MT)-PCR eggs, MT-PCR larvae and larval culture (LC)

Species		Number of positive samples (% prevalence)		
Location	Number of samples	MT-PCR eggs	MT-PCR larvae	LC
*Te. circumcincta*				
Moredun	11	10 (90.90)	7 (63.63)	10 (90.90)
Gent	8	8 (100.00)	8 (100.00)	8 (100.00)
Total	19	18 (94.73)	15 (78.94)	18 (94.73)
*Haemonchus* spp.				
Moredun	11	1 (9.09)	1 (9.09)	1 (9.09)
Gent	8	7 (87.50)	6 (75.00)	6 (75.00)
Total	19	8 (42.10)	7 (36.84)	7 (36.84)
*Trichostrongylus* spp.				
Moredun	11	8 (72.72)	10 (90.90)	6 (54.54)
Gent	8	3 (37.50)	5 (62.50)	5 (62.50)
Total	19	13 (68.42)	15 (78.94)	11 (57.89)
*C. ovina*/*Oesophagostomum* spp.				
Moredun	11	8 (72.72)	8 (72.72)	9 (81.81)
Gent	8	5 (62.50)	3 (37.50)	3 (37.50)
Total	19	13 (68.42)	11 (57.89)	12 (63.15)
*Co. curticei*				
Moredun	11	1 (9.09)	1 (9.09)	2 (18.18)
Gent	8	3 (37.50)	4 (50.00)	2 (25)
Total	19	4 (21.05)	5 (26.31)	4 (21.05)

Also shown are the numbers of samples tested positive for the species/genera of *T. circumcincta*, *Haemonchus* spp., *Trichostrongylus* spp., *Co. curticei* as well as the combined results for *C. ovina* and *Oesophagostomum* spp. and their relative prevalence (%) for the particular locality of Moredun (Scotland) and Ghent (Belgium) as well as for the total dataset

## Discussion

This proof-of-concept study highlighted the utility of the MT-PCR approach for the identification of gastrointestinal nematode infections in naturally infected sheep in Europe. MT-PCR detected every FEC positive sample and demonstrated a perfect agreement compared to microscopy. The highest agreements between MT-PCR (eggs and/or larvae) and LC were obtained at Moredun, Scotland whilst a higher level of discrepancy between the results of the different diagnostic methods was observed in Ghent, Belgium. These differences can be partially explained by the level of experience of the operators at the respective laboratories in regards to the morphological identification of third-stage larvae of small ruminant gastrointestinal nematodes. Moredun, Scotland routinely works with small ruminant parasites and their identification whereas operators at Ghent, Belgium only infrequently deal with the larvae of sheep gastrointestinal nematodes and, therefore, have less experience with their morphological identification, which may have led to a higher incidence of misidentification. This statement is also supported by the finding that observed agreements (i.e. MT-PCR_(e)_ *vs* LC and MT-PCR_(l)_ *vs* LC) were highest for species/genera that are relatively easy to identify morphologically such as *Haemonchus*, *Co. curticei* and the long-tailed larvae of *C. ovina* and *Oesophagostomum* spp. The highest level of discrepancy between the results of the different diagnostic methods employed was observed for the genus *Trichostrongylus,* which is notoriously difficult to differentiate from third-stage larvae of *T. circumcincta*. This is because of the large overlap in the morphometric measurements and the absence of additional distinctive morphological features [6], which probably led to the low level of agreement observed for this genus. These results also reflect our previous experiences with similar comparisons carried out in Australia [7, 19]. Further contributing factors are the incubation temperature employed during LC, which can have an impact on the developmental rate of certain species during LC and thus, may affect the percentage results determined for different species during LC [6, 20, 21]. For the sake of ease of comparison of the results obtained by both diagnostic laboratories, in particular in relation to the LC protocol employed, a common LC protocol could have been prescribed for this study. However, our focus was on comparing the results of the MT-PCR method with the standard LC method as employed by the relevant laboratory, rather than prescribing a protocol which local operators may not be familiar with. Also, the sequencing of all MT-PCR products demonstrated MT-PCR results to be 100% specific, indicating that observed discrepancies between the different diagnostic tests relate to inaccuracies in LC rather than in the MT-PCR method. Interestingly, a higher level of agreement was observed between the results of MT-PCR_(e)_versus LC rather than MT-PCR_(l)_versus LC, which was unexpected but most likely relates to technical issues and/or sample bias (i.e. different subsamples were taken to carry out the different test methods). Furthermore, the volume of sample material used differs significantly between these diagnostic methods (i.e. 250 μl of concentrated eggs for DNA extraction and subsequent MT-PCR as opposed to 20–30 g of faecal matter used in LC method). One factor that probably contributed to these results is that LC samples were not concentrated as much as the eggs (i.e. 250 μl of LC sample was used for DNA isolation but not all larvae in a sample were spun down to create a larval pellet as was done for the eggs). Perhaps this led to a poorer representation of species in the MT-PCR larvae samples because not all larvae were used for DNA isolation. Taken together, these differences are most likely responsible for the observed discrepancies in some samples. Based on our current and past experiences with this MT-PCR platform, we generally prefer the diagnosis based on the amplification of nematode eggs rather than larvae for the following reasons: Eggs can be more easily recovered (i.e. by salt flotation) and their recovery can be integrated into the routine laboratory workflow (e.g. following routine microscopy and egg counts); the use of eggs is faster and more economical in that it does not require extra time and labour and materials for the culture and recovery of larvae; the testing of eggs is likely to be more representative of the different species contributing to a faecal egg count whereas results obtained from the amplification of larval DNA can be influenced by the culture protocol employed for the culture of these larvae which may have altered the proportion of species in the sample (i.e. due to varying rates of development at certain temperatures). However, we also acknowledge that some variation may occur due to the development of eggs during storage and therefore suggest that fresh faecal samples are used for MT-PCR analysis and are stored in an anaerobic environment and/or refrigerated for no longer than 7–10 days before testing [22]. The testing of a larger number of samples would have certainly increased the strength of this comparison but would have gone beyond the scope of this study. Nevertheless, this proof-of-concept study clearly confirmed the high performance of the sheep parasite MT-PCR panel and its ability to specifically detect and identify key species of sheep gastrointestinal parasites. This study also showed that specific and sensitive results can be obtained from a variety of sample types (i.e. eggs and/or larvae) and from different environments and/or countries. Although we haven’t yet tested samples from southern European countries there is a substantial amount of research published from various authors around the world which demonstrates ITS to be a reliable marker for the specific identification of trichostrongylid nematodes. However, further studies to confirm this hypothesis will be required in the future. Because of the relatively small sample size as well as the collection of samples during different times of the year prevalence values recorded for individual genera/species during this study are unlikely to be representative for the given geographic locality or country of origin. However, they do represent an interesting snapshot of the species/genera infecting sheep in the UK and Belgium at the time of sampling. For example, MT-PCR was able to determine a higher prevalence of *H. contortus* in sheep in Belgium compared to Scotland, which would be expected because *Haemonchus* generally prefers warmer climates. The most frequently detected nematode species were *T. circumcincta* and *Trichostrongylus* spp. which is also in accordance with the known epidemiology of these species and in agreement with previous studies [23]. Additionally, the MT-PCR allows for differentiation to species level for *C. ovina* and *Oesophagostomum* spp. with high precision, which would otherwise be difficult if not impossible by morphology of the third-stage larvae. Further advantages of the MT-PCR approach include that following DNA extraction, most of the process is automated and does not require detailed knowledge of molecular diagnostic techniques or livestock parasitology. Also, all MT-PCR reagents are provided in standardized volumes and specifications, are colour-coded and can be traced and easily recorded with specific batch numbers allowing for enhanced Quality Control. These features allow for high consistency in results, and significantly save time, labour and the technical know-how required to carry out sensitive and specific diagnosis and enable direct comparison of diagnostic results between different laboratories and/or countries.

## Conclusions

Collectively, based on the results presented here, as well as our experience with this MT-PCR platform over the past four years, MT-PCR has shown to be a powerful diagnostic tool that offers equivalent or improved diagnostic performance compared to routine diagnostic techniques of microscopy and LC. MT-PCR represents an ideal tool for routine identification of parasitic nematodes in sheep as well as being used as a research tool to carry out comprehensive epidemiological investigations or to assist the diagnosis of drug resistant nematodes, in conjunction with routine faecal egg count reduction testing. Also the ability to use any source of nematode DNA (i.e. eggs, larvae and adult worms) makes this MT-PCR platform a highly versatile tool that can be used to e.g. identify nematode species from pasture larval samples, from gastrointestinal contents or even from encysted tissue stages. However, more research will be required to develop appropriate protocols for such applications. The availability of such an advanced molecular diagnostic tool is increasingly important in monitoring the spread of drug resistance in key species of nematodes infecting domestic livestock and to monitor changes in parasite prevalence and distribution due to climatic changes. With appropriate modifications to the protocol and the specific primers used for diagnosis, this MT-PCR platform could also be used for the diagnosis of other livestock pathogens, for example, gastrointestinal nematodes of cattle (manuscript submitted), trematodes such as *Fasciola hepatica*, protozoans like *Giardia*, *Cryptosporidium* and *Eimeria* or even blood-borne parasites as well as a wide range of other animal, human or environmental pathogens.

## Additional file

Additional file 1: Figure S1. Percentage results for *T. circumcincta*, *Haemonchus* spp., *Trichostrongylus* spp., *Co. curticei* and the combined results for *C. ovina* and *Oesophagostomum* spp. as determined by the three different diagnostic methods used and for each sample. Shown are the results for all samples tested during this study, including samples from Belgium and Scotland. (PDF 332 kb)

## Abbreviations

Ct: Cycle threshold
DNA: Desoxyribonucleic acid
Epg: Eggs per gram of faeces
FEC: Faecal egg count
ITS2: Second internal transcribed spacer
LC: Larval culture
MT-PCR: Multiplexed-tandem PCR
