# Supplementary material for: Multiplexed-tandem PCR for the specific diagnosis of gastrointestinal nematode infections in sheep: an European validation study
Source: Parasit Vectors. 2017 May 8;10:226. doi: 10.1186/s13071-017-2165-x (PMC5422907; doi:10.1186/s13071-017-2165-x)

Ghent samples

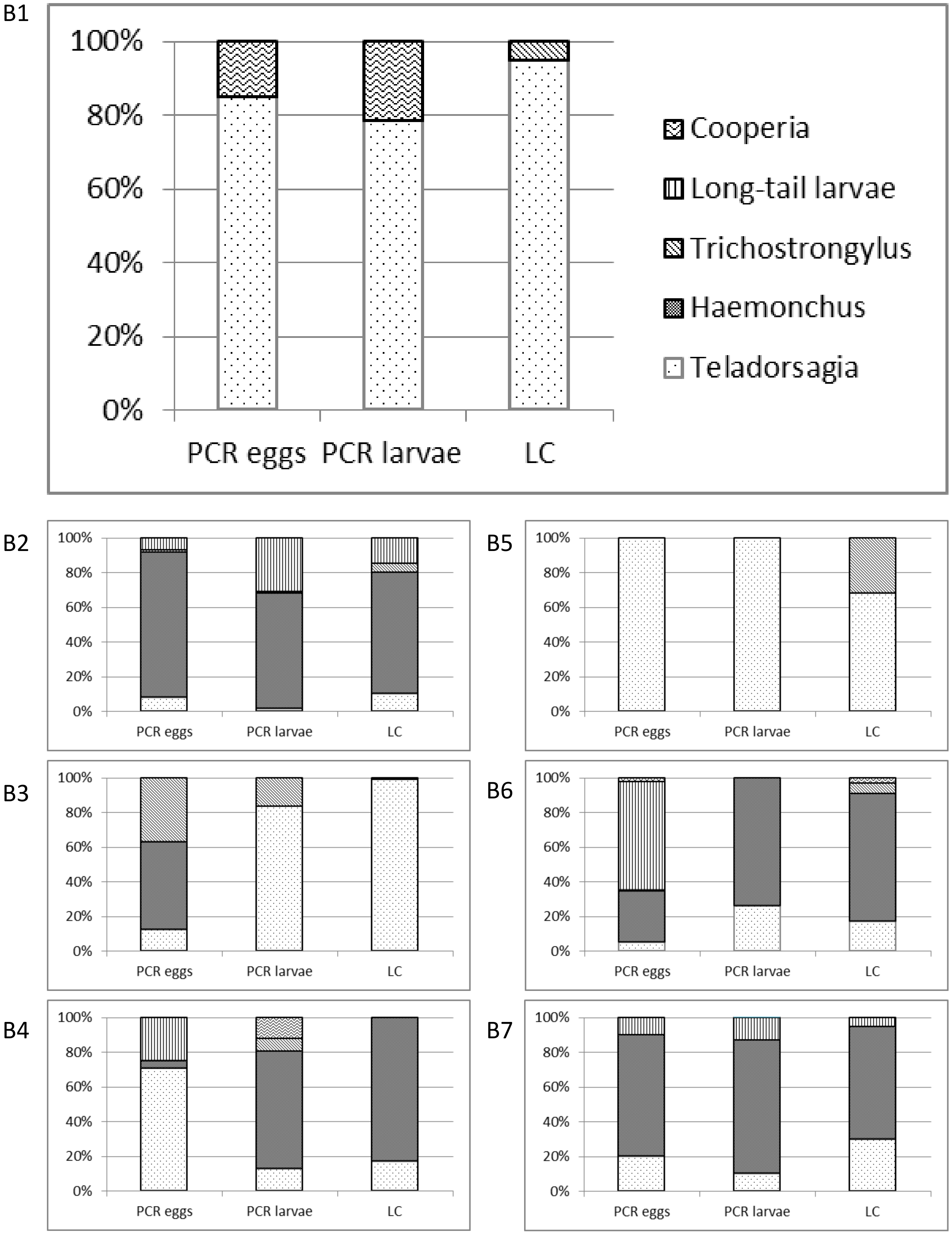

Ghent samples - continued

B8

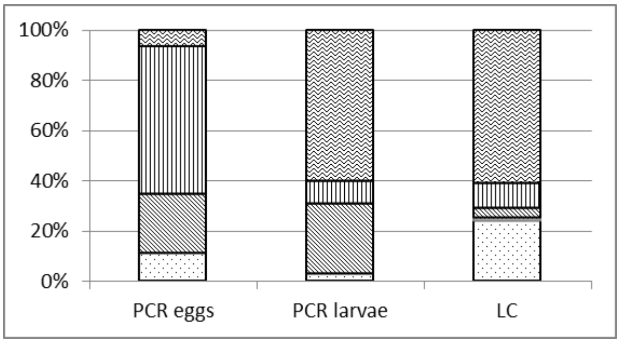

Moredun samples

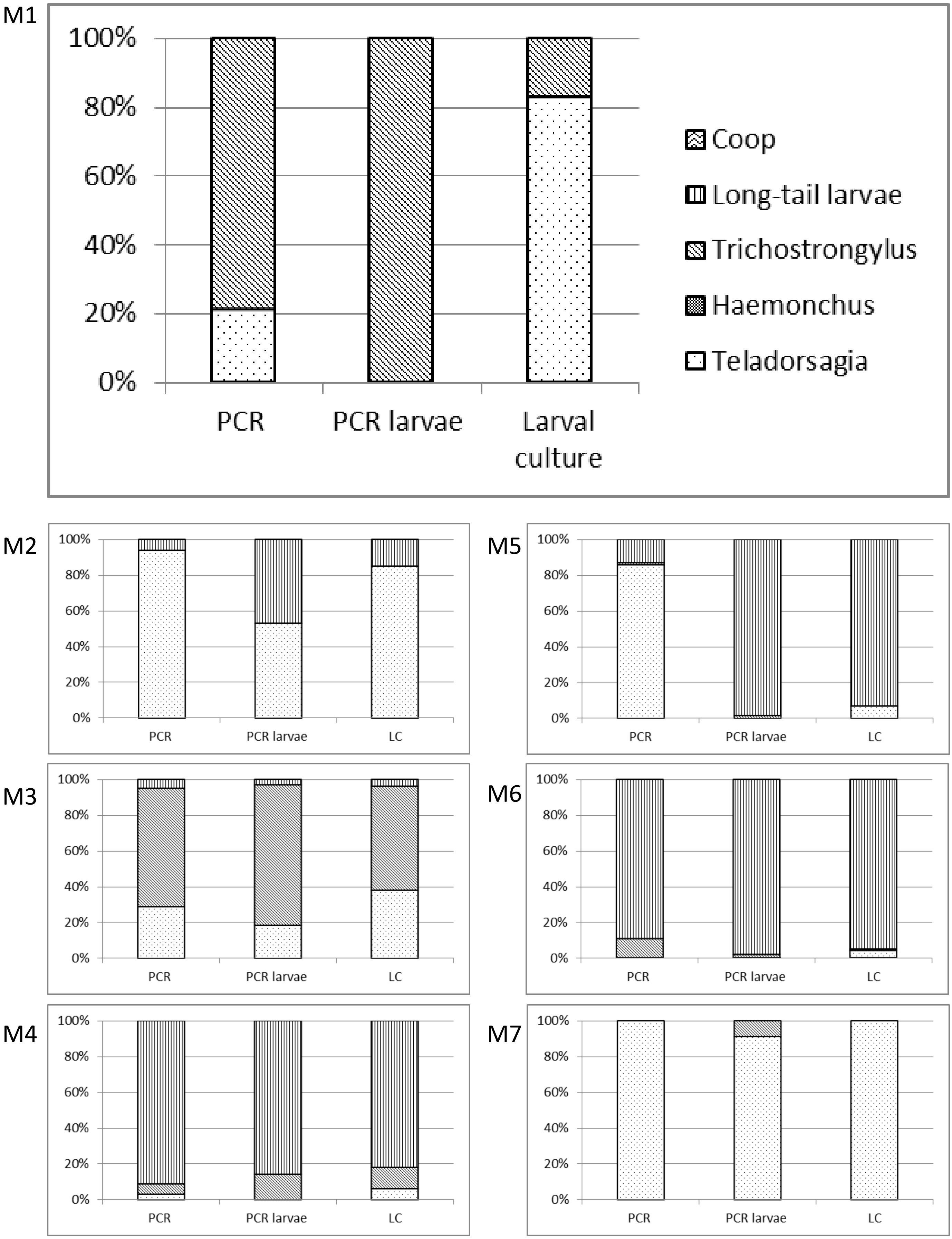

Moredun samples - continued

M8

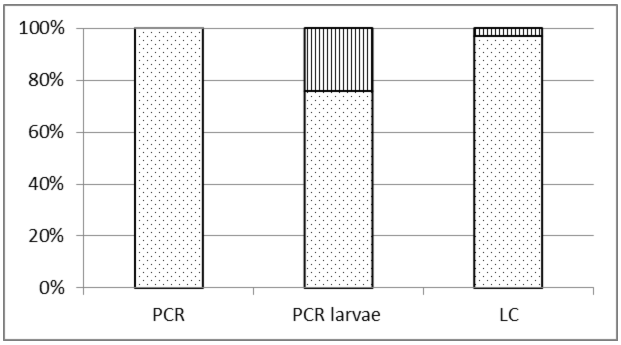

M9

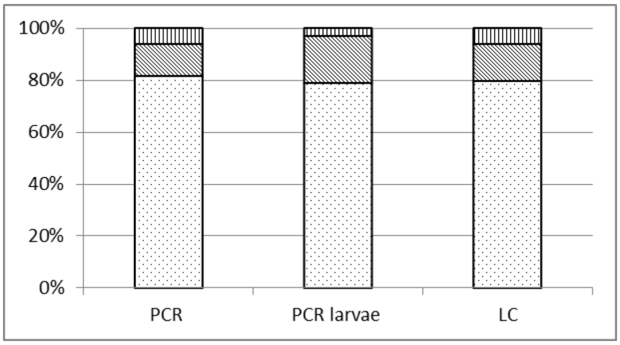

M10

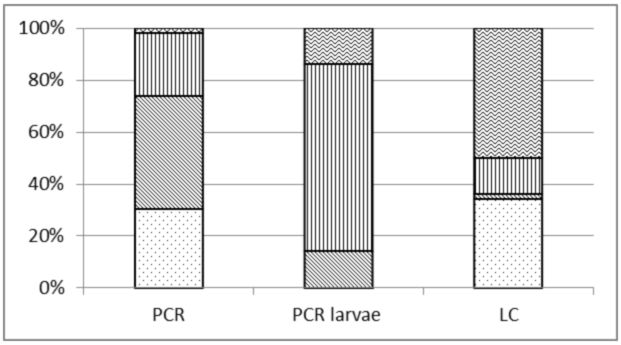

M11

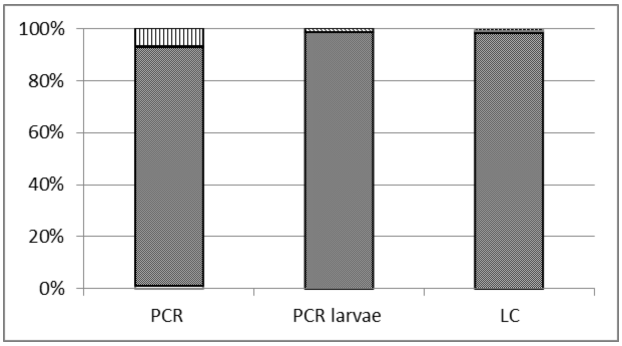

Supplement: Additional file 1: Figure S1. — Percentage results for T. circumcincta, Haemonchus spp., Trichostrongylus spp., Co. curticei and the combined results for C. ovina and Oesophagostomum spp. as determined by the three different diagnostic methods used and for each sample. Shown are the results for all samples tested during this study, including samples from Belgium and Scotland. (PDF 332 kb) [file 13071_2017_2165_MOESM1_ESM.pdf]
